# The effect of thermal cycling on the thermal and chemical stability of paraffin phase change materials (PCMs) composites

**DOI:** 10.1617/s11527-024-02556-y

**Published:** 2024-12-24

**Authors:** Ahmad Wadee, Pete Walker, Nick McCullen, Veronica Ferrandiz-Mas

**Affiliations:** 1 Centre for Integrated Materials, Processes and Structures (IMPS), University of Bath, Bath, BA2 7AY UK; 2 Centre for Climate Adaptation and Environment Research (CAER), University of Bath, Bath, UK; 3 Centre for Regenerative Design and Engineering for a Net Positive World (RENEW), University of Bath, Bath, UK

**Keywords:** Phase change material, Cement, Gypsum plaster, Thermal reliability, Thermal cycling, Latent heat storage, Degradation

## Abstract

This paper is the first study to present the long-term performance of a gypsum and cement plasters which can be used to retrofit existing buildings and reduce their energy consumption. It is comprised of high energy storage loaded granules, known as composite PCMs or form-stable PCMs (FSPCMs), containing three types of organic phase change materials (PCM), with phase change transitions between 18 °C and 25 °C. PCMs are effective thermal energy storage systems as they improve thermal comfort of occupants in buildings by reducing temperature fluctuations. As PCMs will undergo many phase transitions throughout their normal life cycle, the effects of thermal cycling on their long-term stability and performance are important considerations in their selection. The limited understanding on the long-term stability and potential for degradation of PCMs has restricted wider use of these materials in the construction sector. In this research, cement mortar and gypsum plaster specimens were subjected to 700 thermal cycles using an environmental chamber. After cycling, experimental results revealed a reduction of latent heat in the solidification process by up to 23% for the pure PCMs and up to 57% for the PCM loaded granules. However, once the PCMs had been incorporated into either the gypsum plaster or cement mortars, there was no significant reduction in the thermal conductivity or the specific heat capacity of these materials. Thermal cycling did not decrease the effectiveness of PCM composites, and so increasing their potential for wider acceptance of these products and use by the construction industry. This will aid the retrofitting of existing low energy efficient buildings to achieve Net-Zero targets.

## Introduction

Reducing energy use from buildings is one of the biggest challenges globally. One method to aid this reduction is the wider use of phase change materials (PCMs) within buildings as thermal energy storage (TES) solutions. PCMs absorb and release energy during their phase transitions with temperature changes. Latent heat is stored within the specific transition temperature of the PCM [1]. A major advantage of PCMs is their buffering of temperature fluctuations in buildings, providing more stable and comfortable indoor conditions. This however leads to the constant thermal cycling of the PCM as temperatures vary diurnally and seasonally. It is therefore important that the PCM does not degrade as a result of these thermal cycles to ensure longevity in performance. The feasibility of implementing PCMs as latent heat storage within building materials is highly dependent on the long-term chemical and thermal stability of the PCM and the building material. A PCM is reliable if it is thermally and chemically stable after repeated heating and cooling cycles [2]. Ideally, there should be no change to the latent heat or the onset or offset values of the PCM over a long period of continuous thermal cycling.

PCMs are characterised into three categories: organic, inorganic and eutectic mixture, where the melting and solidification temperature of the PCM is lower than its components. For this study organic paraffins were selected; paraffin wax is nontoxic, chemically stable and has a high latent heat capacity [3].

The incorporation of PCMs within building materials is a challenging task, mainly due to potential leakage when PCMs are in liquid state, reduced thermal conductivity of the PCM and low heat storage capacity [3]. Several studies have proposed various methods to incorporate PCMs into construction materials [4–6]. These methods include direct incorporation, where the PCMs are directly mixed with the construction material, and microencapsulation of PCM using an inert organic or synthetic polymer as enclosure shell [7]. However, both of these methods have the risk of leaking when the PCM is in liquid form [8]. To overcome these issues, Wadee et al. developed a novel PCM-loaded granule, using vacuum impregnation to force paraffin PCMs into the pores of aerated concrete granules (ACG) coated with sodium silicate and CEM I 52.5 R powder [9]. This can be referred to as a composite PCM. The term composite PCM is used here to describe PCM retained within a supporting material. The composite PCM can be obtained by immersion, allowing the PCM to be absorbed into the pores of the supporting material by capillary action or vacuum impregnation, forcing the PCM into the pores using vacuum. The immersion method is simple, however the retention capacity of the PCM within the supporting material is relatively low. For the immersion method, longer soaking times leads generally to a greater amount of PCM being impregnated in the LWA [10]. However, any PCM adhering to the surface could be problematic to the mechanical properties when incorporating into a construction material. To increase the retention capacity a vacuum method can be used. Depending on the type of supporting material utilised there are different types of composite PCMs. The smaller powders, such as graphite powder, silica fume, bentonite, diatomite and kaolin, being referred to as form-stable or shape stabilised PCMs (SSPCMs). While larger granules, which make use of lightweight aggregates as supporting materials, referred to as LWA-PCMs. There are several features which must be considered when selecting a supporting material for the PCM to be incorporated into, including porosity, pore structure and chemical compatibility [11]. One method of analysing the porosity and pore structure of a supporting material is mercury intrusion porosimetry (MIP). This involves the intrusion of mercury into the pores of a solid material under increasing pressure. This technique allows for the determination of parameters including pore size distribution, and total pore volume [12].

Using impregnated granules, as opposed to direct incorporation or microencapsulation offer greater potential to incorporate PCMs into several construction materials, including bricks, plaster, cement, or concrete [9, 13–17]. The novel PCM-loaded granules were then incorporated into cement mortar and gypsum plaster to compare the thermal performance of the materials [9, 18]. Whilst some past studies [19, 20] have investigated the thermal stability of PCM impregnated lightweight aggregates—demonstrating stability after up to 1000 thermal cycles—there are no previous studies on the long-term thermal and chemical stability on gypsum or cement plasters which incorporate paraffin impregnated aggregates. Research into the thermal and chemical stability of the PCMs incorporated into building materials is essential to understand their long-term performance and their suitability for wider use in the built environment.

This paper aims to demonstrate the suitability as retrofitting material of novel cement mortars and gypsum plasters containing paraffin based PCMs with phase change transition between 18 °C and 25 °C by analysing their potential for degradation over 700 cycles. This was done by testing the thermal and chemical performance of both PCMs and PCMs-composites using differential scanning calorimetry (DSC), Fourier transform inferred spectroscopy (FT-IR) and thermal analysis using the transient plane source (TPS) method [21–23]. To the best of the authors knowledge, this paper is the first to report the thermal and chemical stability of PCMs based gypsum plaster, which is essential for broader acceptance of these type of materials by the construction industry.

## Materials and methods

### Phase change materials

Three organic paraffin PCMs were used in this study, with melting points between 18 °C and 25 °C, selected based on thermal comfort criteria [24, 25]. The PCMs, supplied by Rubitherm Technologies gmbh, Germany [3], were Rubitherm RT18HC (18 °C), RT22HC (22 °C) and RT25HC (25 °C). The physical properties of each PCM material, obtained from the manufacturers data sheet is displayed in Table 1.

**Table 1:** Physical properties of Rubitherm RT18HC, RT22HC and RT25HC [3]

Property	Rubitherm RT18HC	Rubitherm RT22HC	Rubitherm RT25HC
Density, solid phase	0.88 kg/l	0.76 kg/l	0.88 kg/l
Density, liquid phase	0.77 kg/l	0.70 kg/l	0.77 kg/l
Specific heat capacity	2 kJ/kg K	2 kJ/kg K	2 kJ/kg K
Thermal conductivity	0.2 W/(m K)	0.2 W/(m K)	0.2 W/(m K)
Volume expansion	12.5%	12.5%	12.5%
Freezing temperature	19–17 °C	23–20 °C	26–22 °C
Melting temperature	17–19 °C	20–23 °C	22–26 °C

### PCM loaded granules

Aerated concrete granules (ACGs) were selected to manufacture, form-stable, PCM-loaded granules because of their high porosity. A vacuum impregnation method was used to manufacture the PCM loaded granules. The percentage of PCM within the PCM loaded granules were calculated to be 37.8% for RT18HC, 38.1% for RT22HC and 38.6% for RT25HC [9]. Once filled with the PCM, the granules were coated using CEM I 52.5 R powder and sodium silicate to limit subsequent PCM leakage. To determine the PCM retention of each coating the PCM loaded granules were exposed to a constant temperature of 50 °C for a period of 500 h. 10 g of the PCM loaded granules was placed into a 50 mm diameter container lined with a sponge to collect any PCM leaked; the final mass of the PCM loaded granules was then measured and the total mass loss calculated [9]. The coating material successfully reduced the amount of PCM leakage by 20.8% for the RT18HC loaded granules, 16.8% for the RT22HC loaded granules, and 13.8% for the RT25 loaded granules. The manufacturing process and characterisation of the PCM loaded granules is presented in further detail in a previous study by the authors [9].

### PCM Cement composites

Mortar specimens were manufactured according to BS EN 196-1 [26]. Volume percentages between 10 and 50% of PCM loaded granules were combined with Portland limestone cement CEM II/A-L 32.5 R, potable water and standard silica sand. The specimens were prepared with a binder:sand ratio (by mass) of 1:3 and a water:cement ratio of 1:2 (by mass). Proportions are shown in Table 2. The PCM-loaded granules were added as a replacement for the sand to the total volume of mortar, expressed as the apparent volume of sand (v/v%). The densities of the coated PCM-loaded granules were the same irrespective of the PCM used, and so the mix proportions given are the same for a given replacement percentage of sand [9]. More details about cements mortars manufacturing process and performance can be found in a previous study by the authors [9].

**Table 2:** Mix proportions for manufactured cement mortar specimens where X is the melting temperature for the PCM (18 °C, 22 °C and 25 °C)

Sample	Control	10%RT-X-HC	20%RT-X-HC	30%RT-X-HC	40%RT-X-HC	50%RT-X-HC
Sand (g)	1350	1215	1080	945	810	675
PCM-loaded granules (g)	0	84.7	169.4	254	338.7	423.4

### PCM gypsum composites

PCM loaded granules were combined with gypsum plaster and potable water to manufacture PCM gypsum specimens following the procedures described in BS EN 13279-1 [8]. The water: plaster ratio was kept consistent at 3:5, and mixed together with the PCM loaded granules replacing between 10 and 50% Vol. of gypsum, as shown in Table 3. The densities of the coated PCM-loaded granules were the same for the three types of PCM used, and so like the cement composites the mix proportions were unchanged by PCM material. More details about gypsum mortars manufacturing process and performance can be found in a previous study by the authors [18].

**Table 3:** Mix proportions for manufactured gypsum specimens where X is the melting temperature for the PCM (18 °C, 22 °C and 25 °C)

Sample	Control	10%RT-X-HC	20%RT-X-HC	30%RT-X-HC	40%RT-X-HC	50%RT-X-HC
Gypsum Plaster (g)	1200	1080	960	840	720	600
Water (g)	720	648	576	504	432	360
PCM-loaded granules (g)	0	150	300	450	600	750

### PCM and PCM composite materials characterisation

#### Thermal characterisation

The melting/solidifying temperature range, expressed as the difference between the onset temperature and the offset temperature, and the enthalpy of phase change or latent heat of the pure PCMs and PCMs loaded granules were analysed using Differential Scanning Calorimetry (DSC). The latent heat specifically reflects the PCMs ability to store heat and therefore is indicative of their thermal storage capacity. DSC measures temperatures and heat flows associated with material changes as a function of time and temperature in a controlled environment [6, 7]. The tests were conducted at a ramping rate of 2 °C/min over a temperature range from 0 °C to 40 °C in the heating stage and from 40 °C to 0 °C in the cooling stage, in a nitrogen atmosphere [27]. The results for the phase transition properties (melting/solidification temperatures) and phase change enthalpy or latent heat are presented in Table 4 in the results and discussion section.

**Table 4:** Latent heat capacity, onset and offset temperatures of PCM samples before and after thermal cycling

Material	Melting process			Solidification process		
	Onset temp (°C) ± 0.3 °C	Offset temp (°C) ± 0.3 °C	latent heat (J/g)	Onset temp. (°C)	Offset temp. (°C)	Latent heat (J/g)
RT18HC 0 cycles	19.0	27.9	195.7 ± 5.9	17.0	10.1	202.9 ± 6.1
RT18HC 100 cycles	14.4	28.2	260.4 ± 7.8	16.6	9.2	264.6 ± 7.9
RT18HC 300 cycles	18.8	28	232.9 ± 7.0	16.8	9.6	235.2 ± 7.1
RT18HC 500 cycles	20.7	29.1	219.1 ± 6.6	16.6	8.1	208.3 ± 6.2
RT18HC 700 cycles	12.6	29.5	188.7 ± 5.7	15.8	8.1	172.4 ± 5.2
RT18HC loaded granule 0 cycles	18.0	25.8	75.0 ± 2.2	18.2	11.1	70.7 ± 2.1
RT18HC loaded granule 700 cycles	16.1	23.8	50.2 ± 1.5	16.5	10.2	30.3 ± 0.9
RT22HC 0 cycles	21.2	28.7	118.9 ± 3.6	23.4	14.4	131.5 ± 3.9
RT22HC 100 cycles	21.1	28.7	146.5 ± 4.4	23.0	13.7	114.5 ± 3.4
RT22HC 300 cycles	21.5	29.4	140.3 ± 4.2	21.0	11.5	151.3 ± 4.5
RT22HC 500 cycles	21.5	29.5	122.4 ± 3.7	21.3	10.6	123.6 ± 3.7
RT22HC 700 cycles	21.2	29.4	125.0 ± 3.8	22.0	11.6	125.0 ± 3.8
RT22HC loaded granule 0 cycles	20.8	30.0	50.2 ± 1.5	23.7	15.1	54.7 ± 1.6
RT22HC loaded granule 700 cycles	20.2	28.0	41.0 ± 1.2	23.3	15.7	43.9 ± 1.3
RT25HC 0 cycles	22.2	31.8	158.5 ± 4.8	25.4	17.5	169.5 ± 5.1
RT25HC 100 cycles	21.0	30.3	196.0 ± 5.9	23.9	16.5	202.0 ± 6.1
RT25HC 300 cycles	21.1	30.8	183.5 ± 5.5	24.6	17.4	146.9 ± 4.4
RT25HC 500 cycles	21.0	30.3	168.7 ± 5.1	24.3	16.0	148.8 ± 4.5
RT25HC 700 cycles	22.3	30.1	164.1 ± 4.9	23.8	14.9	130.7 ± 3.9
RT25HC Loaded granule 0 cycles	22.4	30.8	51.3 ± 1.5	25.5	18.0	50.1 ± 1.5
RT25HC Loaded granule 700 cycles	21.7	30.5	55.1 ± 1.7	25.0	17.5	50.3 ± 1.5

Samples were tested at a ramping rate of 2 °C/min over a temperature range from 0 °C to 40 °C

#### Chemical characterisation

**Fig. 1:**
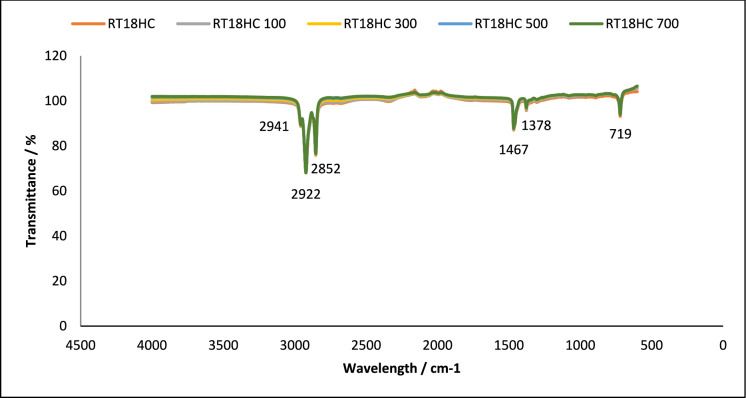
FT-IR of RT18HC, without and after 100, 300, 500 and 700 thermal cycles. Using A PIKE Frontier FT-IR using a Miracle ATR sample compartment, in the wavenumber range of 4000–400 cm^−1^

**Fig. 2:**
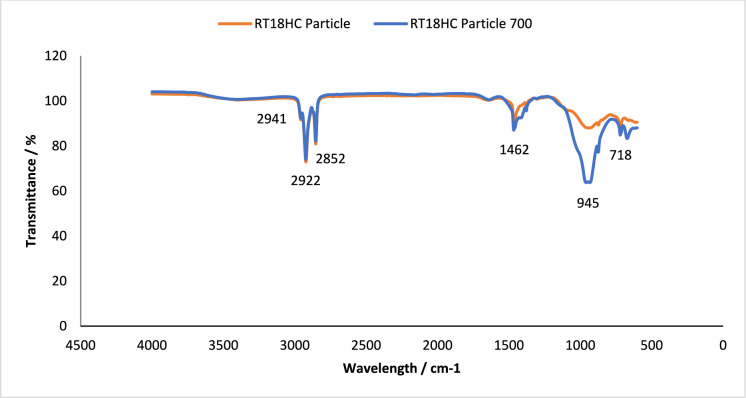
FT-IR of RT18HC loaded granule, without and after 700thermal cycles. Using A PIKE Frontier FT-IR using a Miracle ATR sample compartment, in the wavenumber range of 4000–400 cm^−1^

**Fig. 3:**
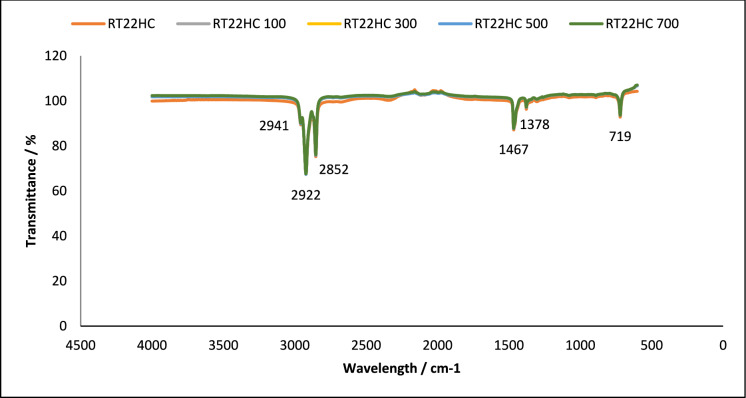
FT-IR of RT22HC, without and after 100, 300, 500 and 700 thermal cycles. Using A PIKE Frontier FT-IR using a Miracle ATR sample compartment, in the wavenumber range of 4000–400 cm^−1^

**Fig. 4:**
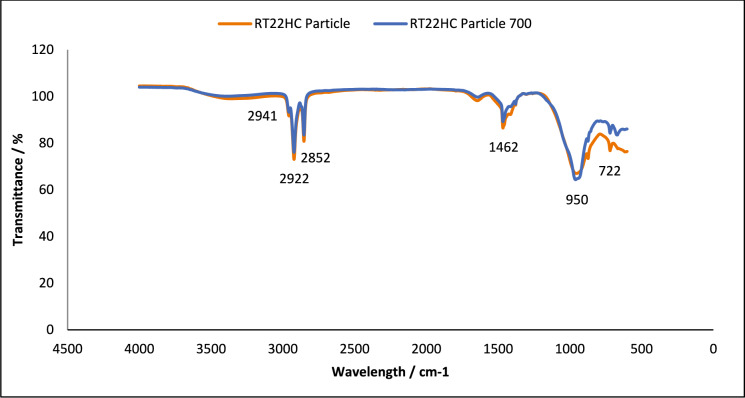
FT-IR of RT22HC loaded granules, without and after 700thermal cycles. Using A PIKE Frontier FT-IR using a Miracle ATR sample compartment, in the wavenumber range of 4000–400 cm^−1^

**Fig. 5:**
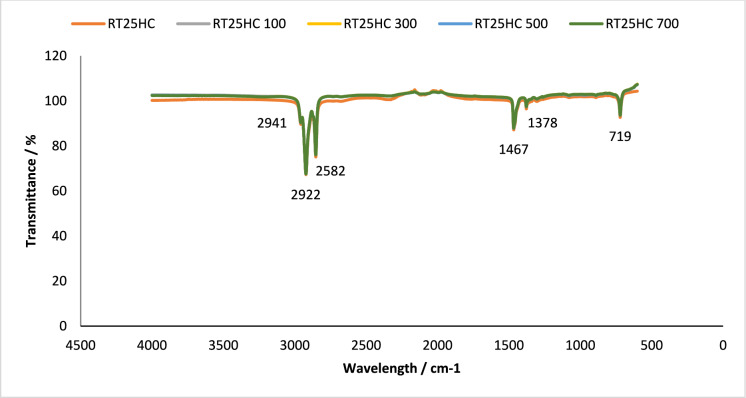
FT-IR of RT25HC, without and after 100, 300, 500 and 700thermal cycles. Using A PIKE Frontier FT-IR using a Miracle ATR sample compartment, in the wavenumber range of 4000–400 cm^−1^

**Fig. 6:**
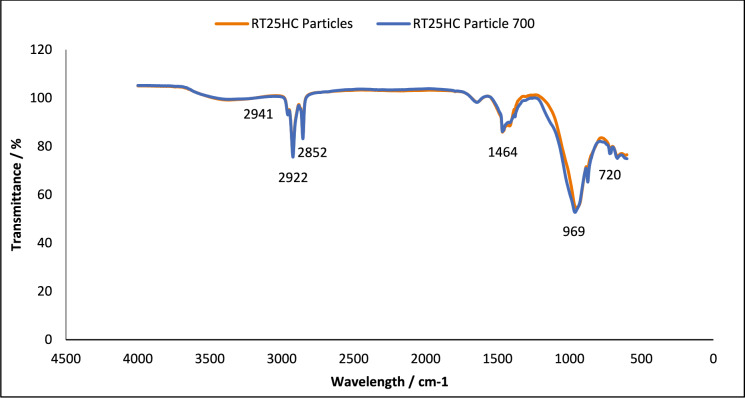
FT-IR of RT25HC loaded granules, without and after 700 thermal cycles. Using A PIKE Frontier FT-IR using a Miracle ATR sample compartment, in the wavenumber range of 4000–400 cm^−1^

The long-term chemical stability of the PCMs and PCM loaded granules were investigated using the Fourier-transform infrared spectroscopy technique (FT-IR). The technique was used to monitor any change in chemical groups before and after thermal cycling. FT-IR passes infrared radiation through a specimen to be absorbed, molecules with different structures produce peaks at different wave numbers [2]. A PIKE Frontier FT-IR using a Miracle ATR sample compartment, in the wavenumber range of 4000–400 cm^−1^ was used for this study. FT-IR was conducted on the pure PCM samples and PCM loaded granules containing RT18HC, RT22HC and RT25HC at 0 and 700 cycles to analyse if there was any change in the chemical stability of the samples. Results are shown in Figs. 1, 2, 3, 4, 5 and 6.

#### Thermal performance

The thermal conductivity and volumetric heat capacity of PCM cement and gypsum composites were tested on 40 mm × 40 mm × 80 mm specimens of using a Hot Disk TPS 500 Thermal Constants Analyser (Thermal Instruments Ltd) [28]. This is a non-destructive test based on the transient plane source technique. A sensor in the shape of a double spiral is sandwiched between two identical specimens. The heat power supplied for a specified time, together with the temperature increase across the specimen, are measured. The relationship between heat supplied and temperature increase provide the basis to calculate the thermal conductivity and volumetric heat capacity [21].

#### Thermal Cycling

Thermal cycle tests were performed using a ACS Benchtop climate chamber, model DY SP110, manufactured by Angelantoni Test Technologies [29]. The cycles were performed every hour using the onset and offset temperatures obtained from the DSC analysis for each of the PCM in this study, allowing for a complete phase change to occur. There is no established number of cycles in the literature to test the long-term stability of PCMs and their composites. Several studies have conducted thermal cycling tests from 100 thermal cycles up to 3600 thermal cycles [30–35]. However, these studies were mostly conducted in PCMs rather than in their composites. 700 cycles were selected based on one melt–freeze cycle per day for 300 days within a year, as stated by Sharma et al. [36], to analyse the stability of the PCM materials for a period of over two years. The thermal cycling effect on the thermal and chemical stabilities of the control, and the specimen containing the lowest (10 v/v%) and highest (50 v/v%) amount of PCM loaded granules were analysed after 100, 300, 500 and 700 cycles. All other specimens were tested after 100 and 700 cycles, by conducting DSC, FT-IR and TPS thermal performance tests.

## Results and discussion

### Thermal stability after thermal cycling

#### DSC

Table 4 shows the enthalpy of phase change or latent heat as well as the onset temperature and the offset temperature of PCM samples in both the endothermic (melting) and exothermic (solidification) process. When PCMs are in solid stage and the temperature increase, PCMs absorb energy and melt in an endothermic process. Reversely, when temperature drops, PCM release energy in an exothermic process, solidifying. The observed melting and solidification processes took place over a range of temperatures as shown in Table 4, not at a single temperature as shown in Table 1. This is due to the commercial PCM data presenting the ideal phase change temperature, however in reality PCMs don’t exhibit an ideal behaviour and often have ‘nonidealities’ associated with them such as, hysteresis or supercooling [37]. Changes in the latent heat of the three studied PCMs and PCM loaded granules were measured over the range of 700 thermal cycles.

As shown in Table 4, during thermal cycling, the onset temperature for the pure RT18HC during melting process was reduced from 19.0 °C to 12.6 °C, whilst the offset temperature increased from 27.9 °C to 29.5 °C. The onset temperature in the solidification process for the same PCM decreased from 17.0 °C to 15.8 °C whilst the offset decreased from 10.1 °C to 8.1 °C. In terms of latent heat, there was an initial increase for RT18HC up to 100 cycles followed by a steady decline up to 700 cycles. This meant a reduction in latent heat ability of 4% in the melting process and 15% in the solidification process. Pure RT22HC did not show significant changes in the onset temperature in melting process and stayed constant at 21.2 °C after 700 cycles. Similar situation was observed for the offset temperature, since the change observed from 28.7 °C to 29.4 °C falls within the instrument error. The solidification process showed a reduction in both the onset temperature and the offset temperature of 1.4 °C and 2.8 °C respectively, resulting in a reduction of latent heat of 5%. Similar to RT18HC, RT25HC showed an initial increase in the latent heat after 100 cycles followed by steady decline up to 700 thermal cycles. The initial increase followed by a decrease in the latent heat of the pure PCMs could be an effect of the rapid heating and cooling of the PCM. Overall, the latent heat for RT25HC in melting phase increased by 4% but decreased by 23% in the solidification phase. The changes observed in the onset temperature in the melting reaction was within the error of the instrument but the offset temperature decreased by 1.7 °C. The onset and offset temperatures in the solidification reactions decreased by 1.6 °C and 2.6 °C, respectively. RT22HC was the most stable of the three PCMs tested after thermal cycling. However, even the other two PCMs tested experienced a reduction in latent heat after thermal cycling the PCMs could still be suitable for use in TES [36].

As the aerated concrete does not melt within the temperature range analysed, the amount of PCM within the inert matrix of the composite PCM can be calculated using the difference in enthalpies at 0 thermal cycles and 700 thermal cycles. Table 5 shows the difference in latent heat for each of the PCMs at 0 and 700 thermal cycles compared to the pure PCM. This percentage indicates the amount of PCM present in the PCM loaded granule. The reduction of PCM in the loaded granule after 700 thermal cycles can be attributed to leaking of the PCM as stated in Sect. 2.2 the coating material sodium silicate combined with CEM I 52.5 R reduced leaking significantly compared to no coating, from between 27.9 and 33.0% to between 11.4 and 14.1% for the three PCMs, after 500 h at 50 °C. However, Wadee et al. discussed the need to research additional coating materials to further reduce the amount of PCM leaking form the composite in a previous study [9].

**Table 5:** Percentage mass of PCM within PCM loaded granule

Material	Endothermic (%)	Exothermic (%)
RT18HC loaded granule 0 cycles	39	35
RT18HC loaded granule 700 cycles	26	15
RT22HC loaded granule 0 cycles	43	42
RT22HC loaded granule 700 cycles	35	34
RT25HC 0 loaded granule cycles	33	30
RT25HC loaded granule 700 cycles	35	30

DSC obtained for RT18HC PCM loaded granules showed a reduction in latent heat of 33% in the melting phase and 57% in the solidification phase after 700 thermal cycles. RT22HC loaded granules showed a reduction of 18 and 20% respectively, whilst RT25HC loaded granules show an increase of 7 and 1%. It is evident that during thermal cycling a proportion of PCM has leaked out of the loaded granule leading to further reduction of latent heat storage ability compared to the pure PCM sample. The CEM I 52.5 R powder and sodium silicate coating on the PCM loaded granule did not completely prevent leakage of the PCM, but it was effective in limiting compared to uncoated granules [9].

These results reflect evidence of potential thermal degradation in the pure PCM samples along with leaking of PCM from the PCM loaded granules that resulted in a reduction of latent heat storage ability which could affect the thermal storage properties of the materials containing the PCM loaded granules during use.

#### Chemical structure stability—FT-IR

To assess the effect of thermal cycling on chemical stability of the studied PCM, their chemical structures were analysed using FT-IR spectroscopy. This technique analyses the chemical bonding in a molecule [38]. The results obtained using FT-IR spectroscopy to analyse any chemical degradation over 700 thermal cycles are presented in Figs. 1, 2, 3, 4, 5 and 6. The disappearance or the addition of new peaks can indicate degradation of the PCM [39].

The pure PCMs presented no chemical degradation over 700 cycles (Figs. 1, 3 and 5), as the FT-IR spectrum show identical profiles before and after thermal cycling. All three cycled PCMs show no additional or disappearance of any peaks, indicating chemical stability of the PCMs. Table 6 lists the different peaks observed in the FT-IR spectrum of RT18HC, RT22HC, and RT25HC PCM and their PCM loaded granules [40–42].

**Table 6:** FT-IR spectrum peaks for RT18HC, RT22HC and RT25HC PCM and PCM loaded granules

Wavenumber (cm^−1^)	Group
945–969 cm^−1^	SiO
719	CH_2_ rocking vibration
1464	CH_2_ and CH_3_ deformation vibration
2941, 2922, 2852	CH_2_ and CH_3_ alkyl stretching vibration

Paraffin wax has three characteristic transmission peaks according to studies found in the literature [42]. The peaks at 2941 cm^−1^, 2922 cm^−1^ and 2852 cm^−1^ refer to the alkyl stretching vibrations of CH_2_ and CH_3_. The peaks at 1464 cm^−1^ refer to the deformation vibration of CH_2_ and CH_3_. The peak at 719 cm^−1^ refers to the rocking vibration of CH_2_ [41, 42]. The FT-IR graphs for the pure PCMs indicate that there is no change in the functional groups at specific wavenumbers.

The FT-IR results for the PCM loaded granules shown in Figs. 2, 4 and 6 showed changes in the peaks between wavenumber 600 and 1000 cm^−1^. This, however, is attributed to the coating material as the wide peak between wavenumber 945–969 cm^−1^ is attributed to SiO [43, 44], from the sodium silicate, CEM I 52.5 R powder and aerated concrete granules. As this wide peak is only shown on the PCM loaded granule spectrum, and not the pure PCM spectrum, all three PCMs loaded granules tested are confirmed to be chemically stable.

#### Thermal performance stability

Tables 7 and 8 present the changes in thermal conductivity and heat capacity of the PCM cement and PCM gypsum composites before and after 700 thermal cycles. Thermal cycling has the potential to degrade the thermal stability of a PCM and the composites that contained them. It is therefore necessary to assess the potential for degradation during use as a proof of their performance and suitability as construction materials.

**Table 7:** Effect of thermal cycling thermal conductivity and heat capacity of the RT18HC, RT22HC and RT25HC cement composites, with an increase in percentage replacement of PCM loaded granules

Percentage PCM replacement	Thermal conductivity (W/m K) 0 cycles	Thermal conductivity (W/m K) 700 cycles	Percentage change (%)	Heat capacity (MJ/m^3^ K) 0 cycles	Heat capacity (MJ/m^3^ K) 700 cycles	Percentage change (%)
Control 0%	2.67 (± 0.07)	2.98 (± 0.09)	+ 12%	1.65 (± 0.16)	1.68 (± 0.17)	+ 2
RT18HC 10%	2.39 (± 0.01)	2.32 (± 0.03)	− 3%	1.84 (± 0.08)	2.04 (± 0.08)	+ 11
RT18HC 20%	1.77 (± 0.01)	1.90 (± 0.03)	+ 7%	1.83 (± 0.02)	1.91 (± 0.04)	+ 4
RT18HC 30%	1.54 (± 0.01)	1.90 (± 0.01)	+ 23%	1.90 (± 0.01)	2.00 (± 0.01)	+ 5
RT18HC 40%	1.49 (± 0.01)	1.33 (± 0.02)	− 11%	2.21 (± 0.03)	2.55 (± 0.08)	+ 15
RT18HC 50%	1.10 (± 0.02)	1.08 (± 0.02)	− 2%	2.61 (± 0.08)	2.76 (± 0.10)	+ 6
RT22HC 10%	2.20 (± 0.01)	2.32 (± 0.01)	+ 6%	1.87 (± 0.02)	2.06 (± 0.03)	+ 10
RT22HC 20%	2.20 (± 0.00)	2.12 (± 0.11)	− 4%	1.97 (± 0.01)	1.99 (± 0.15)	+ 1
RT22HC 30%	1.88 (± 0.00)	1.99 (± 0.00)	+ 6%	2.03 (± 0.00)	2.25 (± 0.01)	+ 11
RT22HC 40%	1.43 (± 0.02)	1.64 (± 0.02)	+ 15%	2.05 (± 0.06)	2.34 (± 0.07)	+ 14
RT22HC 50%	1.07 (± 0.06)	1.11 (± 0.04)	+ 4%	2.37 (± 0.23)	2.51 (± 0.12)	+ 6
RT25HC 10%	2.14 (± 0.01)	2.50 (± 0.02)	+ 17%	1.80 (± 0.06)	1.90 (± 0.04)	+ 6
RT25HC 20%	1.99 (± 0.01)	2.11 (± 0.01)	+ 6%	1.93 (± 0.03)	1.98 (± 0.01)	+ 3
RT25HC 30%	1.93 (± 0.01)	1.96 (± 0.01)	+ 2%	1.97 (± 0.01)	2.04 (± 0.10)	+ 4
RT25HC 40%	1.39 (± 0.01)	1.64 (± 0.12)	+ 18%	2.09 (± 0.01)	2.12 (± 0.33)	+ 1
RT25HC 50%	1.40 (± 0.01)	1.32 (± 0.17)	+ 6%	2.30 (± 0.01)	2.27 (± 0.50)	− 1

Thermal conductivity and heat capacity measured using a TPS 500 S with a heating power of 500 mW and a measurement time of 20 s, with the SD shown as a percentage

**Table 8:** Effect of thermal cycling on thermal conductivity and heat capacity of the RT18HC, RT22HC and RT25HC gypsum composites, with an increase in percentage replacement of PCM loaded granules

Percentage PCM replacement	Thermal conductivity (W/m·K) 0 cycles	Thermal conductivity (W/m·K) 700 cycles	Percentage change (%)	Heat capacity (MJ/m^3^·K) 0 cycles	Heat capacity (MJ/m^3^·K) 700 cycles	Percentage change (%)
Control 0%	0.35 (± 0.01)	0.35 (± 0.01)	0%	0.96 (± 0.01)	0.99 (± 0.01)	+ 3
RT18HC 10%	0.39 (± 0.01)	0.38 (± 0.01)	− 3%	1.05 (± 0.03)	1.01 (± 0.01)	− 4
RT18HC 20%	0.39 (± 0.01)	0.39 (± 0.01)	0%	1.12 (± 0.02)	1.12 (± 0.02)	+ 0
RT18HC 30%	0.40 (± 0.01)	0.41 (± 0.01)	+ 3%	1.22 (± 0.01)	1.19 (± 0.01)	− 3
RT18HC 40%	0.41 (± 0.01)	0.43 (± 0.01)	+ 5%	1.35 (± 0.02)	1.16 (± 0.03)	− 14
RT18HC 50%	0.40 (± 0.01)	0.40 (± 0.01)	0%	1.49 (± 0.04)	1.37 (± 0.01)	− 8
RT22HC 10%	0.37 (± 0.01)	0.38 (± 0.01)	+ 3%	1.08 (± 0.01)	1.14 (± 0.01)	+ 6
RT22HC 20%	0.39 (± 0.01)	0.39 (± 0.01)	0%	1.12 (± 0.04)	1.07 (± 0.02)	− 5
RT22HC 30%	0.40 (± 0.01)	0.40 (± 0.01)	0%	1.17 (± 0.03)	1.16 (± 0.01)	− 1
RT22HC 40%	0.42 (± 0.01)	0.44 (± 0.01)	+ 5%	1.44 (± 0.02)	1.40 (± 0.03)	− 3
RT22HC 50%	0.44 (± 0.01)	0.45 (± 0.01)	+ 2%	1.57 (± 0.02)	1.61 (± 0.03)	+ 3
RT25HC 10%	0.34 (± 0.01)	0.36 (± 0.01)	+ 6%	0.99 (± 0.03)	0.91 (± 0.02)	− 8
RT25HC 20%	0.38 (± 0.01)	0.40 (± 0.01)	+ 5%	1.17 (± 0.01)	1.04 (± 0.02)	− 11
RT25HC 30%	0.39 (± 0.01)	0.40 (± 0.01)	+ 3%	1.23 (± 0.02)	1.15 (± 0.02)	− 7
RT25HC 40%	0.43 (± 0.01)	0.43 (± 0.01)	0%	1.52 (± 0.01)	1.52 (± 0.02)	+ 0
RT25HC 50%	0.45 (± 0.01)	0.43 (± 0.01)	− 4%	1.67 (± 0.05)	1.67 (± 0.01)	+ 0

Thermal conductivity and heat capacity measured using a TPS 500 S with a heating power of 100 mW and a measurement time of 80 s, with the SD shown as a percentage

For the cement composites (Table 7), the addition of PCM loaded granules decreased the thermal conductivity in both cases before and after cycles, compared to the control materials without PCM loaded granules. The highest reduction in thermal conductivity compared with the control was for RT22HC at 50% replacement, which thermal conductivities dropped 60% before and 64% after cycles. This corresponded with thermal conductivities of 1.07 ± 0.06 W/m K and 1.11 ± 0.04 W/m K. When comparing the same PCM cement composite before and after 700 cycles, the effect of thermal cycling on the thermal conductivity does not seem to have a pattern. In some cases increased the thermal conductivity after cycling up to 23% (RT18HC 30%) and in others decreased the thermal conductivity up to 11% (RT18HC 40%). However, in all the cases the thermal conductivity was higher than the average range found in the literature for organic PCMs materials, between 0.15 and 0.20 W/m K [15]. In a PCM, a high thermal conductivity is important since increases the rate of heat absorption/release throughout the PCM. This is translated in an increment in the effectiveness at which it can store and release thermal energy, which is vital to obtain a system which fully utilise the latent heat storage ability of the PCM composite. The heat capacity of the cement composites (Table 7) increased when PCM loaded granules were added to them, compared with the control samples without PCMs. The highest increment in heat capacity was observed for the 50% replacement using RT18HC, where the heat capacity increased 58% before cycles and 64% after cycles. This corresponded with heat capacity values of up to 2.61 ± 0.08 MJ/m^3^ K and 2.76 ± 0.10 MJ/m^3^ K. When comparing the effect of thermal cycling on the heat capacity of PCM composites, thermal cycling seemed to have a positive effect, with increments of up to 15% for the cement composites containing RT18HC 40%. Overall, the thermal cycling was not detrimental to the thermal performance of the cement composites containing PCM loaded granules.

Table 8 presents the data for the thermal conductivity and heat capacity before and after cycles for the PCM loaded granules gypsum composites. In most of the cases, thermal conductivity remained the same as the control (without PCM loaded granules) or slightly increased when adding PCM loaded granules, which is beneficial for the overall thermal performance of the PCM composites [15]. The composites with the biggest increment in thermal conductivity were RT25HC 50% before thermal cycling and RT22HC 50% after thermal cycling. For both of them the thermal conductivity increased by 28.6%, and the thermal conductivity value was 0.44 W/m K, higher than the values obtained for reported organic PCMs [15]. As discussed above, an increment in the thermal conductivity is beneficial to increase the effectiveness at which the PCM composite is able to store/release thermal energy. When looking at the effect of thermal cycling on the thermal conductivity of the PCM gypsum composites, the thermal conductivity of these composites remained the ·same or slightly increased, which shows not detrimental effect of the thermal cycling on these materials.

In terms of the heat capacity of the gypsum composites, the results in Table 8 showed an increment when PCM loaded granules were added to the control gypsum. The highest increment in heat capacity of 74% was for the gypsum composite with RT25HC 50% replacement, which heat capacity increased from 0.96 MJ/m^3^ K for the control to 1.67 MJ/m^3^ K. This behaviour proves the benefit of adding PCM loaded granules in the heat storage capacity of the gypsum plaster. When gypsum plaster composites containing the PCM loaded granules were subject to thermal cycling, the heat capacity either increased or remain the same in most of the cases. However, reduction up to 14% were found for some of the composites (i.e. RT18HC 20%). Although these reductions in heat capacity did not significantly affect the thermal performance of the composites. Overall, after thermal cycling, the three studied PCMs showed good thermal stability after being incorporated into cement and gypsum plasters.

## Conclusions

This paper reports for the first time the long-term performance of a gypsum plaster which can be used to retrofit existing buildings and reduce their energy consumption. This study focused on the thermal and chemical stability of three paraffin PCMs with phase transition temperatures between 18 °C and 25 °C, which are suitable temperatures to achieve thermal comfort in buildings. The PCMs tested have potential for long term use in TES. The thermal and chemical stability of the three studied PCMs, after 700 thermal cycles were compared with the results without thermal cycling. The following conclusions are drawn from the investigation:

The DSC thermal analysis show that thermal cycling does have an effect on the latent heat and the onset and offset temperatures of all three PCMs in both the melting and solidification phases. The results show RT22HC to be the most stable with a 5% increase in latent heat in the melting phase and a 5% decrease in the solidification phase. However, none of the changes to the latent heat of the pure PCMs are considered significant or limit potential use of the materials in the built environment.

Thermal cycling did have an effect on the PCM loaded granules according to the DSC data, with the latent heat of RT18HC granule reducing by 33 and 57%, RT22HC granules reducing by 18 and 20%, whilst RT25HC increased by 7 and 1% in the melting and solidification phases. DSC analysis of the pure PCM and FT-IR data, demonstrated that the significant reduction in latent heat for RT18HC and RT22HC is due to PCM leakage from the coating material during thermal cycling.

FT-IR analysis did not show any chemical degradation of the three PCMs studied after thermal cycling, as there were no changes to the chemical structure of the paraffins. However, after 700 thermal cycles, there were changes in the peaks between wavenumber 600–1000 cm^−1^ for RT18HC loaded granules and RT22HC loaded granules, indicating that the PCM leaked from the coating material. Despite the changes, the three PCMs have shown good potential for use in TES applications.

After incorporation of PCM loaded granules into cement and gypsum there was no significant impact of thermal cycling to the thermal conductivity and heat capacity of the PCM composite materials. Even though DSC results showed that leakage of the PCM loaded granules significantly reduced its latent heat ability, after incorporation into cement and gypsum any leaked PCM material during thermal cycling was minimised by the cement and gypsum.

This study has demonstrated paraffin based PCMs to be chemically and thermally stable and therefore suitable for use in TES applications, with potential to significantly reduce energy usage in buildings. This paper fills the gap of lack of studies in long term thermal and chemical stability of both cement and gypsum based PCMs composites, which will increase the construction industry acceptance of these materials for their application in building retrofitting.
